# Circulating Heme Oxygenase-1: Not a Predictor of Preeclampsia but Highly Expressed in Pregnant Women Who Subsequently Develop Severe Preeclampsia

**DOI:** 10.1155/2018/6035868

**Published:** 2018-09-30

**Authors:** Valéria C. Sandrim, Mayara Caldeira-Dias, Heloisa Bettiol, Marco Antonio Barbieri, Viviane Cunha Cardoso, Ricardo Carvalho Cavalli

**Affiliations:** ^1^Department of Pharmacology, Institute of Biosciences, São Paulo State University (UNESP), Botucatu, São Paulo, Brazil; ^2^Center of Toxicological Assistance (CEATOX), Institute of Biosciences, São Paulo State University (UNESP), Botucatu, São Paulo, Brazil; ^3^Department of Pediatrics, Faculty of Medicine of Ribeirao Preto, University of Sao Paulo, Ribeirao Preto, Sao Paulo, Brazil; ^4^Department of Obstetric and Gynecology, Faculty of Medicine of Ribeirao Preto, University of Sao Paulo, Ribeirao Preto, Sao Paulo, Brazil

## Abstract

Preeclampsia is the major cause of maternal and fetal deaths worldwide. Circulating biomarker concentrations to predict preeclampsia must be determined. Therefore, the objective was to evaluate heme oxygenase-1 (HO-1) concentration in both plasma and urine samples from pregnant women before the development of preeclampsia and to identify a potential biomarker for preeclampsia development. We performed a case-control study nested in a prospective study cohort at University Hospital of the Ribeirao Preto Medical School, University of São Paulo (HCFMRP-USP), Ribeirao Preto, Brazil. Of 1400 pregnant women evaluated at 20–25 weeks of gestation, 460 delivered in hospitals outside our institution. Of 940 pregnant women who completed the protocol, 30 developed preeclampsia (cases, 14 cases of severe preeclampsia and 16 cases of mild preeclampsia). Healthy pregnant women (controls, *n* = 90) were randomly selected from the remaining 910 participants. HO-1 concentration was evaluated in plasma/urine samples by using a commercial enzyme-linked immunosorbent assay kit. We found similar HO-1 levels in the plasma and urine for case and control groups. In the subgrouped preeclampsia, lower plasma HO-1 levels were found in mild compared with severe preeclampsia. We conclude that plasma HO-1 levels were not altered at 20–25 weeks of gestation before the manifestation of preeclampsia symptoms. Pregnant women who subsequently develop severe preeclampsia show higher expression of HO-1. This may be indicative of important underlying pathophysiologic mechanisms that differentiate between mild and severe preeclampsia and may possibly be related to a higher prooxidative status even before the development of clinical symptoms.

## 1. Introduction

Preeclampsia is a syndrome characterized by hypertension associated with proteinuria or other systemic signs and is considered a major causal factor for maternal and fetal morbidity [1]. Predicting preeclampsia is important to reduce complications for the mother and fetus and to decrease the number of deaths related to the syndrome. Although there have been many efforts in the past, few markers are available for early diagnosis [2, 3]. The potential biomarkers evaluated are circulating antiangiogenic factors, soluble fms-like tyrosine kinase-1 (sFLT-1), and placental growth factor (PIGF) [4]. Both are angiogenesis markers because sFLT-1 (the soluble receptor of VEGF, vascular endothelial growth factor) has antiangiogenic properties since it inhibits VEGF and PIGF binding to the receptor, consequently reducing the pathway signaling of these molecules [5].

Besides (anti) angiogenic factors, other biomarkers have been evaluated to predict preeclampsia early, including homocysteine [6], myeloperoxidase [7], placental protein 13 (PP13, a member of the galectin family) [8], and pregnancy-associated protein A (PAPP-A) [9].

Heme oxygenase-1 (HO-1) is an enzyme that cleaves heme-producing bilirubin and carbon monoxide (CO), which promotes cell protection, through antioxidant, antiapoptotic, and anti-inflammatory properties [10]. HO-1 expression is inducible and promoted by heme, proinflammatory cytokines, endotoxin, and reactive oxidative stress (ROS) [11]. Recently, increasing interest has been given to HO-1 in preeclampsia by mainly studying the placental tissue [12–15], and interestingly, George et al. [16] demonstrated that the induction of HO-1 attenuates placental ischemia-induced hypertension in a rat model of preeclampsia, whereas inhibition of HO-1 activity increases the mean blood pressure [17].

Despite intense recent research regarding HO-1 in preeclampsia, the evaluation of circulating HO-1 levels to predict preeclampsia is missing to date. Therefore, this study aimed at exploring HO-1 concentration in both plasma and urine samples from pregnant women before the development of preeclampsia and at identifying a potential biomarker for the development of preeclampsia.

## 2. Material and Methods

### 2.1. Study Design and Population

This nested cohort study is part of a larger observational prospective study with a main objective of assessing new risk factors for preterm birth and the impact of perinatal indicators in fetal and infant growth in two different Brazilian cohorts: Ribeirao Preto City, Sao Paulo, and Sao Luis City, Maranhao BRISA Cohort [18]. Data described here are only associated to the Ribeirao Preto cohort. Pregnant women were recruited at hospitals and healthcare units during their first-trimester prenatal visit (until 20 weeks of gestation with ultrasound confirmation) and were invited to visit the University Hospital of the Ribeirao Preto Medical School, University of São Paulo (HCFMRP-USP), Ribeirao Preto, Brazil, to participate in the study. Clinical evaluation and blood collection were performed during the second trimester of gestation (at 20–25 weeks).

**Figure 1:**
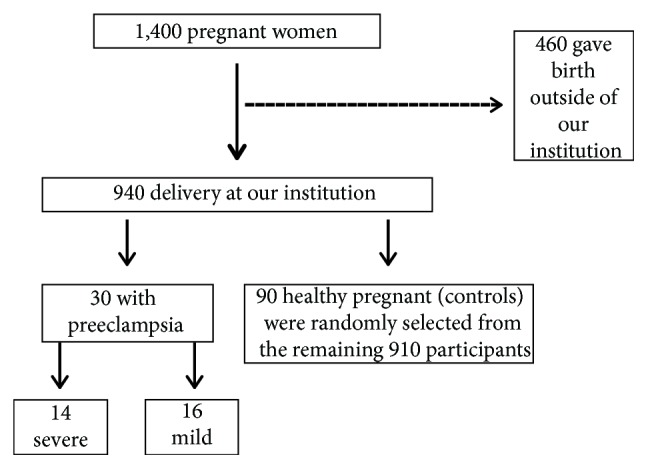
Enrolment of study participants.

The participants were recruited as follows (Figure 1): of 1400 pregnant women evaluated at the HCFMRP-USP at 20–25 weeks of gestation, 460 delivered in hospitals outside our institution. Of 940 pregnant women who completed the protocol, 30 developed preeclampsia. Only healthy pregnant women and singleton pregnancy (controls, *n* = 90) were randomly selected from the remaining 910 participants. This was because the variation in HO-1 concentrations is higher than that in the concentrations of the biomarker previously evaluated. This study was approved by the Institutional Review Board of the HCFMRP-USP (reference 4116/2008, approved date November 11, 2008). All participants provided written informed consent. All studies complied with the principles of the Declaration of Helsinki.

## 3. Results

General clinical parameters for controls (pregnant women who remained healthy throughout pregnancy) and for cases (who later developed mild or severe preeclampsia) are summarized in Table 1. No differences were observed in maternal age, body mass index, and gestational age at sampling. The systolic blood pressure was elevated in the severe case group, and the diastolic blood pressure was also increased in both severe and mild case groups compared with the control group. Moreover, patients with severe preeclampsia had lower newborn weight and gestational age at delivery compared with both controls and patients with mild preeclampsia.

**Figure 2:**
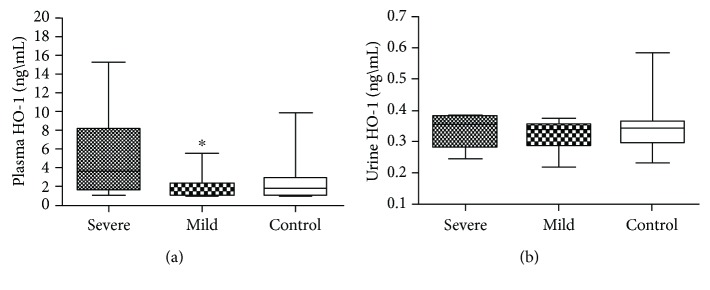
Plasma (a) and urine (b) HO-1 concentration from pregnant healthy during gestation (control, *n* = 90) and women who subsequently developed severe (*n* = 14) and mild (*n* = 16) preeclampsia. Significant differences were found in plasma HO-1 between mild and severe preeclampsia (medians [25th–75th centiles]: 1.38 ng/mL [1.16–2.26] vs. 3.62 ng/mL [1.68–8.16], *P* < 0.05). Urine levels were similar among groups (0.33 ± 0.05, 0.32 ± 0.04, and 0.34 ± 0.6, respectively, severe, mild, and control). Boxplot indicates median [min−max]. Comparison of concentration among groups was by Kruskal-Wallis followed by Dunn's or ANOVA 1-way followed by Tukey's multiple comparisons test.

We found similar plasma HO-1 levels in the case and control groups (medians [25th–75th centiles], 1.69 ng/mL [1.19–4.17] vs. 1.83 ng/mL [1.22–2.90], *P* = 0.54), and similar levels were also noted in the urine (0.33 ± 0.05 ng/mL vs. 0.34 ± 0.06 ng/mL; *P* = 0.31, Figure S1). Interestingly, when we subgrouped mild or severe preeclampsia, we found significant differences in plasma HO-1 levels among the groups (Figure 2(a), respectively, *P* = 0.03). The mild case group showed lower plasma HO-1 levels compared with severe case group (medians [25th–75th centiles]: 1.38 ng/mL [1.16–2.26] vs. 3.62 ng/mL [1.68–8.16], *P* < 0.05), and similar levels compared with the control (1.82 ng/mL [1.22–2.90], *P* > 0.05). Similar urine HO-1 levels were found among the groups (Figure 2(b)). In addition, we correlated plasma HO-1 levels with clinical parameters, and significant correlations were lacking (Table S1).

## 4. Discussion

### 4.1. Main Findings

Pregnant women who will develop preeclampsia present similar plasma and urine HO-1 levels compared with pregnant women who are healthy throughout pregnancy. Therefore, neither plasma nor urine HO-1 level is a good biomarker to predict preeclampsia at 20–25 weeks of gestation. However, interestingly to the pathophysiology point, we observed lower plasma HO-1 concentration in the group that will develop mild preeclampsia compared with that in the group who will develop severe preeclampsia, suggesting an important mechanism of pregnancy regulation in preeclampsia.

### 4.2. Strengths and Limitations

Strengths include the recruitment of a high number of participants in an unselected general population, prospective cohort, and blood collection before the development of preeclampsia. The limitations of our study include not collecting sequential blood mainly after the development of preeclampsia; the small number of severe and mild preeclampsia cases, increasing the chance of alpha type error; measurement of plasma HO-1 level, and this represents a sum of all HO-1 tissue production; not measuring HO-1 level before 20 weeks of gestation, which may show us other results; and when the study was conducted.

### 4.3. Interpretation

To the best of our knowledge, this is the first study to show HO-1 levels in pregnant women before clinical onset of preeclampsia; therefore, we were unable to compare our results with others. However, it is important to note that even after developing preeclampsia, few studies reported circulating HO-1 in preeclampsia compared with healthy controls [13, 19]. Interestingly, Erdemli et al. observed higher serum HO-1 levels in preeclamptic women [76.7 ng/mL (23.4–445.7), *n* = 33] at 27–34 weeks of gestation compared with normotensive pregnant women [55.9 ng/mL (3.7–354.3), *n* = 43] [20]. Vitoratos et al. measured serum HO-1 level at 30–34 weeks of gestation at the time of diagnosis [19]. They recruited 10 healthy pregnant women, 9 women with mild preeclampsia, and 12 women with severe preeclampsia and found that higher serum HO-1 levels were found in women with severe preeclampsia (5.5 ± 1.5 ng/mL) compared with the other groups (3.0 ± 0.7 and 3.1 ± 1.6 ng/mL, respectively, mild and healthy pregnant). Subsequently, these data corroborate with ours and demonstrate that molecular mechanism could be activating HO-1 expression in severe preeclampsia. Eide et al. [13] verified the serum HO-1 levels in preeclampsia (*n* = 9) and healthy controls (*n* = 8) and found significant increased levels in preeclampsia pregnant women (3.1 ± 1.3 vs. 1.9 ± 0.5 ng/mL). In this study, the age of gestation that the serum was collected was unclear; however, the serum may have been probably collected at delivery because the decidua was also collected. Studies have shown that HO-1 is increased in placental tissues from preeclamptic women [21–23]; however, it is not clear if this alteration could be reflected in the plasma. Erdemli et al. suggested that circulating levels of HO-1 could represent a “leakage” of this enzyme from other tissues, similar to the presence of liver enzymes in the circulation [20].

The important differences among mild and severe preeclampsia are interesting and may be associated with the underlying mechanism that can differentiate these two subtypes of preeclampsia. One mechanism may involve higher levels of free iron in severe preeclampsia (136 ± 50 *μ*g/dL) compared with mild cases (98 ± 48 *μ*g/dL) and healthy controls (73 ± 31 *μ*g/dL) as demonstrated by Serdar et al. [24]. Moreover, because free iron is a potent oxidant, both mechanisms can activate HO-1 expression in pregnant women that subsequently develop severe preeclampsia. We also know that preeclampsia, mainly severe, is involved an unbalance of placental pro (oxidative) species; hence, it is possible that these species, by activation of the transcription mechanism of HO-1, may be related to higher HO-1 levels in severe compared with mild preeclampsia. Other studies have shown different levels of biomarkers between early- and late-onset preeclampsia [25], showing differences in pathophysiological mechanisms. In early-onset preeclampsia, biomarkers such as FLT-1, ICAM (intercellular adhesion molecule 1), and NAD(P)H (nicotinamide adenine dinucleotide phosphate) oxidase are increased compared in late-onset preeclampsia [26]. Although the classification of early (<34 weeks) or late (≥34 weeks) preeclampsia is based on gestational age at diagnosis or delivery and not to severity of the clinical symptoms, pregnant women with early preeclampsia present clinical onset characteristic of severe preeclampsia and late preeclampsia present the onset of mild preeclampsia.

## 5. Conclusion

In conclusion, plasma HO-1 levels were not altered at 20–25 weeks of gestation before the manifestation of clinical symptoms. However, pregnant women who subsequently develop severe preeclampsia present higher expression of HO-1, and this may indicate an important underlying pathophysiologic mechanism that can differentiate mild and severe preeclampsia, possibly related to higher prooxidative status even before the development of clinical symptoms. Therefore, an identification of a biomarker related to severe preeclampsia may help in monitoring these patients, decreasing mortality and lesions in target-organ lesions.

## Figures and Tables

**Table 1:** General characteristics of patients enrolled in the study.

Parameters	Control	Case	
		Mild	Severe
*n*	*90*	*16*	*14*
Age (years)	25.9 ± 5.9	27.1 ± 5.6	29.0 ± 5.8
BMI (kg/m^2^)	28.2 ± 5.3	29.4 ± 5.0	28.3 ± 5.3
Skin color (white/not white)	42/48	8/8	9/5
SBP (mmHg)	108.0 ± 11.2	114.8 ± 9.8	119.3 ± 13.4^∗^
DBP (mmHg)	65.9 ± 7.1	71.9 ± 6.5^∗^	75.9 ± 10.8^∗^
Nulliparous (%)	32 (35.0)	7 (43.7)	4 (28.6)
GA at sampling (weeks)	23.4 ± 1.5	23.6 ± 1.4	23.1 ± 1.1
GA at delivery (weeks)	39.5 ± 1.6	38.8 ± 1.1	35.1 ± 4.3^∗^^#^
NBW (g)	3336.2 ± 456.2	3370.0 ± 520.9	2364.0 ± 1011.0^∗^^#^
APGAR score 1 (1 min) < 7	15	2	4
APGAR score 2 (5 min) < 7	1	0	2

Information in this table is referent to the time of blood and urine collection before preeclampsia diagnosis, except for GA at delivery and NBW. Data as mean ± SD or *n* (percentage of total). BMI, body mass index; SBP, systolic blood pressure; DBP, diastolic blood pressure; PE, preeclampsia; GA, gestational age; NBW, newborn weight. ^∗^ vs. control; ^#^ vs. mild case.

## Data Availability

The data used to support the findings of this study are available from the corresponding author upon request.

## Supplementary Materials

Supplementary Materials Levels of plasma (A) and urine (B) HO-1 between case and control subjects. Figure S1: plasma (A) and urine (B) HO-1 concentration from pregnant healthy during gestation (control, *n* = 90) and women who subsequently developed preeclampsia (case, *n* = 30). No significant differences were found in plasma between case and control of plasma (medians [25th–75th centiles]: 1.69 ng/mL [1.19–4.17] vs. 1.83 [1.22–2.90], respectively; *P* = 0.54) neither urine (0.33 ± 0.05 ng/mL vs. 0.34 ± 0.06 ng/mL, respectively; *P* = 0.31). Boxplot indicates median [min−max]. Comparison between groups was Student's *T*-test. Table S1: correlations between general characteristics and HO-1 concentration in plasma samples from controls and mild and severe cases.
