# Supplementary material for: Circulating Heme Oxygenase-1: Not a Predictor of Preeclampsia but Highly Expressed in Pregnant Women Who Subsequently Develop Severe Preeclampsia
Source: Oxid Med Cell Longev. 2018 Sep 30;2018:6035868. doi: 10.1155/2018/6035868 (PMC6186313; doi:10.1155/2018/6035868)
Supplement: Supplementary Materials — Levels of plasma (A) and urine (B) HO-1 between case and control subjects. Figure S1: plasma (A) and urine (B) HO-1 concentration from pregnant healthy during gestation (control, n = 90) and women who subsequently developed preeclampsia (case, n = 30). No significant differences were found in plasma between case and control of plasma (medians [25th–75th centiles]: 1.69 ng/mL [1.19–4.17] vs. 1.83 [1.22–2.90], respectively; P = 0.54) neither urine (0.33 ± 0.05 ng/mL vs. 0.34 ± 0.06 ng/mL, respectively; P = 0.31). Boxplot indicates median [min−max]. Comparison between groups was Student's T-test. Table S1: correlations between general characteristics and HO-1 concentration in plasma samples from controls and mild and severe cases. [file 6035868.f1.docx]

SUPPORTING INFORMATION

**Circulating heme oxygenase-1: not a predictor of preeclampsia, but highly expressed in pregnant women who subsequently develop severe preeclampsia**

Valéria C. SANDRIM^1,2*^, Mayara CALDEIRA-DIAS^1^, Heloisa BETTIOL^3^, Marco Antonio BARBIERI ^3^, Viviane Cunha CARDOSO ^3^, Ricardo Carvalho CAVALLI^4^

^1^ Departament of Pharmacology, Institute of Biosciences, São Paulo State University (UNESP), Botucatu, São Paulo, Brazil

^2^ CEATOX – Center of Toxicological Assistance - Institute of Biosciences, São Paulo State University (UNESP), Botucatu, São Paulo, Brazil

^3^ Department of Pediatrics, Faculty of Medicine of Ribeirao Preto, University of Sao Paulo, Ribeirao Preto, Sao Paulo, Brazil.

^4^ Departament Of Obstetric and Gynecology, Faculty of Medicine of Ribeirao Preto, University of Sao Paulo, Ribeirao Preto, Sao Paulo, Brazil


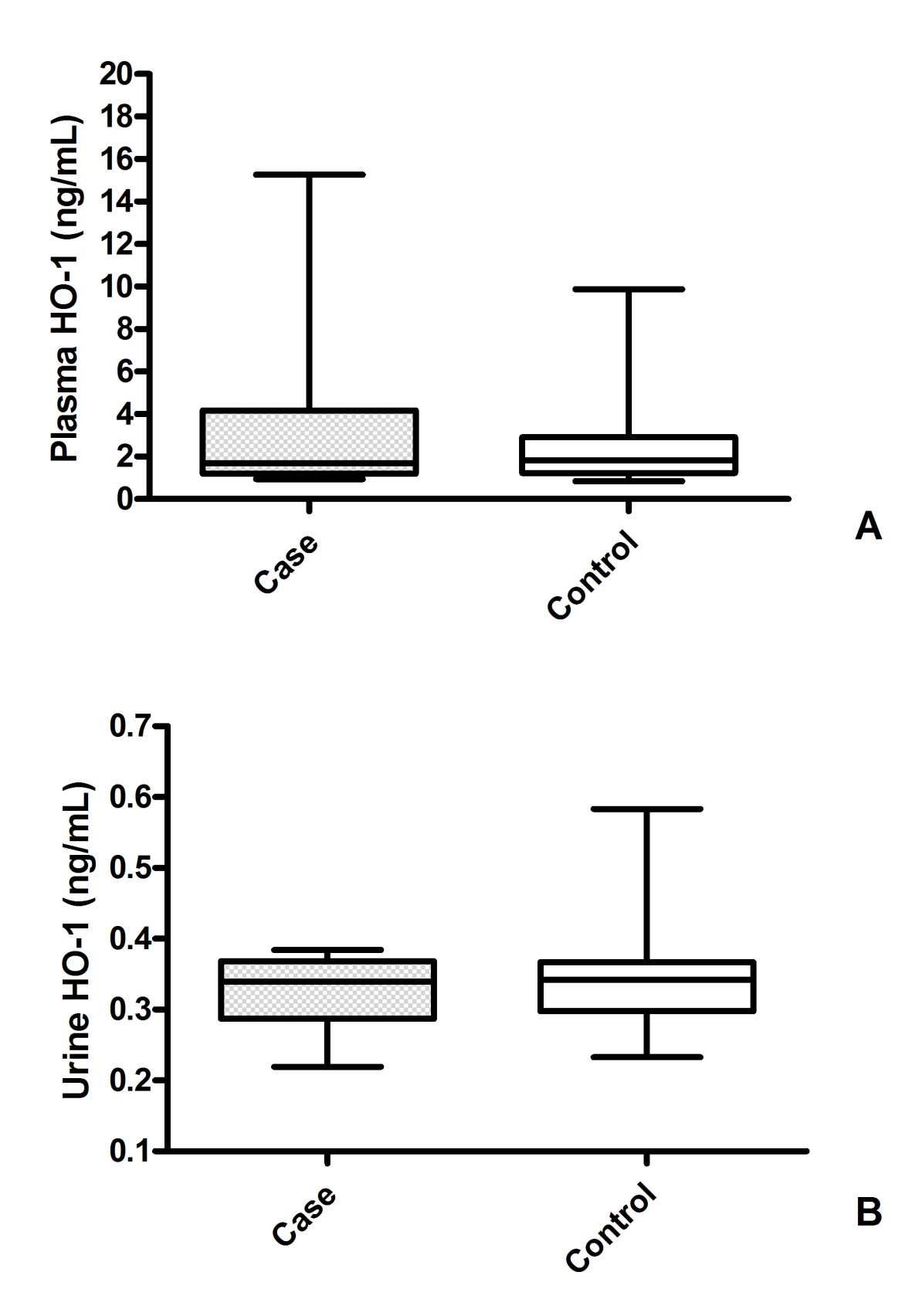


**Figure S1** –Plasma (A) and urine (B) HO-1 concentration from pregnant healthy during gestation (control, n=90) and women who subsequently developed preeclampsia (case, n=30). No significant differences were found in plasma between case and control of plasma (medians [25th–75th centiles]: 1.69 ng/mL [1.19-4.17] *vs* 1.83 [1.22-2.90], respectively; *P=0.54*) neither urine (0.33±0.05 ng/mL *vs* 0.34±0.06 ng/mL, respectively *P=0.31*). Boxplot indicates median [min−max]. Comparison between groups was Student´s T-test.

**Table S1.** Correlations between general characteristics and HO-1 concentration in plasma samples from controls, mild and severe cases

|  | **Severe (Case)** | **Mild (Case)** | **Control** |
| --- | --- | --- | --- |
| SBP (mmHg) | -0.24 (0.42) | -0.04 (0.87) | -0.05 (0.62) |
| DBP (mmHg) | 0.00 (0.97) | 0.13 (0.62) | -0.09 (0.37) |
| BMI (Kg/m^2^) | -0.01 (0.95) | -0.31 (0.23) | -0.19 (0.08) |
| Maternal Age | -0.22 (0.46) | -0.10 (0.70) | -0.12 (0.26) |
| NBW (g) | 0.35 (0.23) | -0.18 (0.49) | -0.12 (0.24) |
| Urine levels | -0.02 (0.94) | -0.49 (0.06) | -0.13 (0.23) |

Data as correlation coefficient (*P* value); SBP, systolic blood pressure; DBP, diastolic blood pressure; BMI, body mass index GA, gestational age; NBW, newborn weight.
